# Cryptic collagen IV promotes cell migration and adhesion in myeloid leukemia

**DOI:** 10.1002/cam4.203

**Published:** 2014-02-12

**Authors:** Amanda J Favreau, Calvin P H Vary, Peter C Brooks, Pradeep Sathyanarayana

**Affiliations:** 1 Center for Molecular Medicine, Maine Medical Center Research InstituteScarborough, 04074, Maine; 2 The Graduate School of Biomedical Science and Engineering, University of MaineOrono, 04469, Maine; 3 Department of Medicine, Tufts University School of MedicineBoston, Maine

**Keywords:** AKT, AML, microenvironment, collagen, DDR1

## Abstract

Previously, we showed that discoidin domain receptor 1 (DDR1), a class of collagen-activated receptor tyrosine kinase (RTK) was highly upregulated on bone marrow (BM)-derived CD33+ leukemic blasts of acute myeloid leukemia (AML) patients. Herein as DDR1 is a class of collagen-activated RTK, we attempt to understand the role of native and remodeled collagen IV in BM microenvironment and its functional significance in leukemic cells. Exposure to denatured collagen IV significantly increased the migration and adhesion of K562 cells, which also resulted in increased activation of DDR1 and AKT. Further, levels of MMP9 were increased in conditioned media (CM) of denatured collagen IV exposed cells. Mass spectrometric liquid chromatography/tandem mass spectrometry QSTAR proteomic analysis revealed exclusive presence of Secretogranin 3 and InaD-like protein in the denatured collagen IV CM. Importantly, BM samples of AML patients exhibited increased levels of remodeled collagen IV compared to native as analyzed via anti-HUIV26 antibody. Taken together, for the first time, we demonstrate that remodeled collagen IV is a potent activator of DDR1 and AKT that also modulates both migration and adhesion of myeloid leukemia cells. Additionally, high levels of the HUIV26 cryptic collagen IV epitope are expressed in BM of AML patients. Further understanding of this phenomenon may lead to the development of therapeutic agents that directly modulate the BM microenvironment and attenuate leukemogenesis.

## Introduction

Leukemia stem cells (LSCs) have been shown to occupy niches in the endosteal and sinusoidal areas of bone marrow (BM) where signals from these niches are known to promote survival, maintenance, and fate specifications of LSCs 1. Characterization of the intrinsic and extrinsic components of this niche signaling is critical for understanding the pathophysiology and development of niche-targeted therapeutics for myeloid leukemia. Previously, we showed for the first time that discoidin domain receptor 1 (DDR1), a class of collagen-activated receptor tyrosine kinase (RTK), was highly upregulated on BM-derived CD33+ leukemic blasts of acute myeloid leukemia (AML) patients 2. Further, this study demonstrated miR-199b targets DDR1 and levels of miR-199b and DDR1 are inversely correlated 2. Herein as DDR1 is a class of collagen-activated RTK, we attempt to understand the role of native and remodeled collagen IV in BM microenvironment and its functional significance in leukemic cells.

Proteolytic remodeling of the collagenous extracellular matrix (ECM) has been shown to result in generation of functionally important cryptic collagen epitopes, which under normal physiologic conditions, are masked in its quaternary structure 3. Exposure of a cryptic site on collagen IV, previously characterized as HUIV26, was shown to be exposed within the ECM of angiogenic blood vessels and malignant tumors 3. Exposure of the HUIV26 epitope in vitro*,* resulted in reduced alpha1beta1 integrin binding and gain of alphaVbeta3 binding in solid phase-binding assays 3. Importantly, targeting of HUIV26 epitope via a monoclonal antibody resulted in significant inhibition of angiogenesis, primary tumor growth, and experimental metastasis of B16F10 melanoma in vivo 4. Here, we investigated the role of native and remodeled collagen IV in BM microenvironment and its functional significance in leukemic cells. Also, we examined the relative cryptic collagen IV epitope HUIV26 in BM samples of AML patients.

## Methods

### Cell Culture

K562 cells were cultured in Iscove's Modified Dulbecco's Medium (IMDM) with 10% fetal bovine serum (FBS) and 1× PSF (Penicillin, Streptomycin, and Fungizone = Amphotericin B).

### Migration and adhesion assays

Briefly, K562 cells (1 × 10^5^) were harvested and suspended in serum-free media with either soluble native or denatured collagen IV (10 *μ*g/mL). The cells were allowed to migrate through Costar Transwell Membranes (8.0-*μ*m pore size) to complete media (10% FBS) for 4 h. At 4 h, the inner chamber was removed and three different 75 *μ*L aliquots of the outer chamber media was examined on Moxi Z Cell Counter (ORFLO Technologies, Hailey, ID) to determine the migrated cell counts. These treatments were performed in triplicate. For adhesion assays, briefly, 48-well plates were coated with native or denatured collagen IV (10 *μ*g/mL) at 4°C for 18 h. K562 cells (1 × 10^5^) were harvested and suspended in adhesion buffer (IMDM containing 1 mmol/L MgCl_2_, 0.2 mmol/L MnCl_2_, and 0.5% bovine serum albumin), added to wells, and allowed to attach for 2 or 4 h at 37°C. Nonattached cells were removed by washing, and attached cells were fixed and stained with crystal violet. Cell adhesion was quantified by counting number of stained nuclei. These treatments were performed in triplicate.

### Flow cytometry

For DDR1 protein analysis, cells were washed in phosphate-buffered saline (PBS) and resuspended in 200 *μ*L of PBS. Cells were then incubated with 5 *μ*L of anti-DDR1 antibody conjugated with paraffin embedded (PE, PE anti-human CD167a, Biolegend, San Diego, CA; cat #334005) and 1.5 *μ*L of ChromPure Rat IgG Whole Molecule (Jackson ImmunoResearch #012-000-003; Jackson ImmunoResearch Laboratories, Inc., West Grove, PA) for 1 h shaking at 4°C. Following incubation, cells were washed and resuspended in 500 *μ*L of PBS and analyzed on flow using FACScalibur (BD Biosciences, San Jose, CA).

### siRNA knockdown

K562 cells were transfected with control and DDR1 siRNA (Thermo Scientific siControl ON-TARGETplus CONTROLpool catalog #D-001810-10-05 and siDDR1 ON-TARGETplus SMARTpool catalog #L-003111-00; Fisher Scientific, Pittsburgh, PA) using Invitrogen's Liopfectamine LTX with PLUS Reagent (catalog #15338-100; Invitrogen, Life Technologies, Grand Island, NY) following the protocol provided by Life Technologies for K562 cells: http://www.lifetechnologies.com/us/en/home/references/protocols/cell-culture/transfection-protocol/transfecting-plasmid-dna-into-k562-cells-using-lipofectamine-ltx-reagent.html.

### Western blot and enzyme-linked immunosorbent assay

K562 cells were treated for 15 min with either soluble native or denatured collagen IV (10 *μ*g/mL) prior to cell lysis. Cells were lysed in M-PER mammalian protein extraction lysis buffer (Thermo Scientific, Cat # 78501) containing Halt protease and phosphatase inhibitor cocktail (Thermo Scientific, Cat # 78442) and cleared lysates were assayed for protein content, denatured, electrophoresed, transferred to polyvinylidene difluoride membranes, and blotted. Primary antibodies for both phosphorylated and total AKT, MAPK, and STAT5 as well as total DDR1 and *β*-tubulin were obtained from Cell Signaling (Cell Signaling Technology, Inc., Danvers, MA). Primary MMP-9 antibody for conditioned media (CM) analysis was obtained from Millipore. Horseradish peroxidase-conjugated antibodies and enhanced chemiluminescence reagents were as described previously 5. Briefly, CM was obtained from K562 cells cultured in serum-free media with either soluble native or denatured collagen IV (10 *μ*g/mL) for 24 h. Media was collected and concentrated on ice and at 4°C using Millipore Centrifugal Filter Units (0.2 *μ*m). For ELISA, K562 cells were treated for 15 min with either soluble native or denatured collagen IV (10 *μ*g/mL) prior to cell lysis. The PathScan® Phospho-DDR1 (panTyr) Sandwich ELISA Kit #7863 (Cell Signaling) was utilized per manufacturer's instructions. Cell lysates were analyzed via Western blot for total DDR1 levels. These treatments were performed in triplicate.

### Mass spectrometry

Conditioned media as previously described in the Western Blot methods was utilized for analysis. For isotope-coded affinity tag (ICAT) tandem mass spectrometry (MS/MS), the CM were concentrated by ultracentrifugation, labeled, and purified using the Cleavable ICAT Reagent Kit for Protein Labeling (Applied Biosystems, Life Technologies, Grand Island, NY), and analyzed with a tandem quadrupole time-of-flight mass spectrometer (QSTAR; MDS-SCIEX, AB SCIEX, Framingham, MA) as described by Koleva et al. 6. Analysis of mass spectrometric data was conducted using ProteinPilot software program (Life Technologies).

### Construction of tissue microarray and immunohistochemistry analysis

A custom-made tissue microarray (TMA) for *n* = 24 PE BM samples of AML patients and *n* = 10 control/normal samples was constructed using the services of US Biomax, Inc. (Rockville, MD). The array was in a single core per case format. Tissue array sections were mounted on the positively charged SuperFrost Plus glass slide. The TMA sections were cut at 5 micron thickness. Individual cores were 1.5 mm in diameter and spaced 0.25 mm apart. Anti-human collagen IV antibody suitable for PE-IHCs was obtained from R&D Systems (R&D Systems, Inc., Minneapolis, MN) and HUIV26, monoclonal antibody against cryptic collagen IV epitope was generated as previously described 7. To optimize antibody concentration for immunohistochemistry (IHC) on paraffin-embedded tissue array slides, a pilot experiment was performed using serial dilutions of the primary antibody on testing slides to determine the optimal working condition. For IHC, standard protocol was followed. Briefly, antigen retrieval was performed with 1× antigen retrieval solution (Target Retrieval solution, S-1699; DAKO Cytomation, Dako North America, Inc., Carpinteria, CA). For detection, ImmPRESS™ Reagent [anti-Goat Ig (peroxidase)] (Vector Laboratories, Inc., Burlingame, CA) and DAB (3,3'-diaminobenzidine) as substrate chromogen (DAKO Cytomation) was used. Hematoxylin was used for counterstaining cell nuclei. IHC-stained slides were scanned and the total positive cell numbers and intensity of anti-Collagen IV or anti-HUIV26 staining was computed and measured by ImageScope from Aperio Scanning System (Leica Microsystems Inc., Buffalo Grove, IL). In addition, an independent pathologist also performed manual scoring of the intensity levels.

### Statistical analysis

The significance of experimental results was determined by Student *t*-test unless otherwise noted.

## Results and Discussion

**Figure 1:**
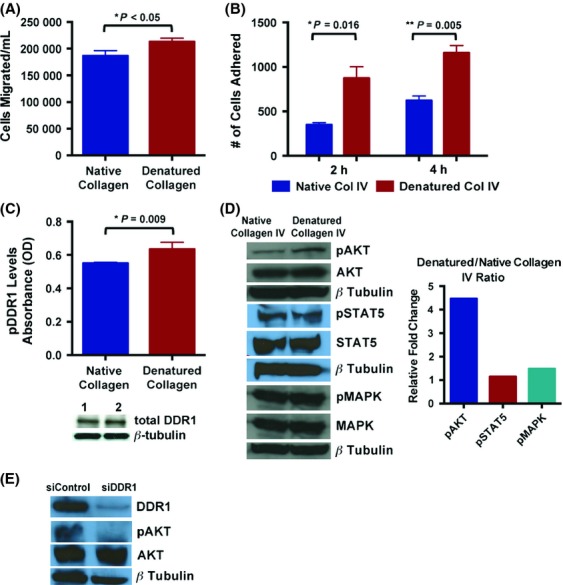
Exposure to cryptic collagen IV significantly increases migration and adhesion potentially via activations of DDR1 and AKT in K562 cells. (A) Transwell migration of K562 cells exposed to either soluble native or heat-denatured collagen IV after 4 h. (B) Cell adhesion assays were performed in K562 cells upon exposure to native or denatured collagen IV. (C) DDR1 phosphorylation levels were examined using PathScan pDDR1 (panTyr) Sandwich ELISA upon exposure to either soluble native or heat-denatured collagen IV. Total DDR1 was assessed in the ELISA lysates (1 = Native Collagen IV, 2 = Denatured Collagen IV). (D) Activation of AKT, STAT5 and MAPK was examined via phosphoblotting. Steady-state levels including *β*-tubulin levels were also assessed (left panel). Phosphoblotting was quantitated using Image J and normalized to total protein and *β*-tubulin to calculate the ratio of phosphorylation between denatured and native collagen exposure (right panel). (E) DDR1 expression was knockdown using siRNA for DDR1. Activation of AKT was examined using phosphoblotting. Total protein levels including *β*-tubulin were also assessed.

Previously, we showed downregulation of DDR1 expression by miR-199b in K562 cells resulted in decreased migration 2. As DDR1 is a collagen-activated RTK and cellular interactions with HUIV26 collagen epitope can regulate cellular motility, we hypothesized that remodeled collagen IV may promote increased migration. Analysis of multiple myeloid leukemia cell lines indicated robust expression of DDR1 on K562 cells (Fig. S1), therefore, this cell line was chosen to further investigate DDR1's role in myeloid leukemia. Exposure of K562 to denatured collagen IV resulted in significantly enhanced migration rates compared to native collagen IV as determined using Corning Costar transwell migration assay (Fig. 1A). Additionally, DDR1 has been previously shown in various malignant cells such as glioma to mediate adhesion to collagen and promote invasion 8. Significantly, K562 indicated preferential adhesion to denatured collagen IV compared to native collagen IV (Fig. 1B). The functional findings of increased migration and adhesion to denatured collagen IV indicate DDR1 and its downstream signaling may be molecularly altered when exposed to denatured collagen IV compared to native collagen IV. Briefly, both DDR1 and DDR2 have been shown to bind various forms of collagen but each has preferential binding specificity such as collagen IV for DDR1 and collagen II and X for DDR2 8. Interestingly, DDRs are thought to have a binding affinity to GVMGFO-motifs as seen with collagens I-III, but the binding affinity for collagen IV is not known, although it does not appear to preferentially bind to these GVMGFO-motifs 9. DDR1 phosphorylation levels, examined via PathScan pDDR1 (panTyr) Sandwich ELISA, were significantly increased upon exposure of denatured collagen IV (Fig. 1C). This could indicate that sites within remodeled collagen IV may have preferential binding affinity compared to native collagen IV motifs and therefore increase DDR1 activity. Downstream DDR1 signaling activates many pathways such as AKT, MAPK, and STAT5 8. Of these downstream signaling molecules, AKT showed increased phosphorylation upon exposure of denatured collagen IV but MAPK and STAT5 were unchanged (Fig. 1D). We then wanted to confirm whether DDR1 directly activates AKT. Knockdown of DDR1 via siRNA demonstrated that silencing of DDR1 significantly attenuates AKT activation (Fig. 1E). With AKT and DDR1 both being known to affect cell proliferation, we examined if there was a change in proliferation upon exposure to native or denatured collagen IV but it was unaffected (data not shown). Activation of AKT has been shown to promote migration of Th1 lymphocytes via CD152/CCL4/CCR5-mediated PI3K activation 10. Further, constitutive activation of AKT during B cell maturation lead to increased STAT5 activation and cell migration to the chemokine SDF1*α* 11. Furthermore, hematopoietic stem cells (HSCs) are highly sensitive to AKT/mTOR signaling and importantly; increased activation of AKT in HSCs induces AML at the expense of HSC pool 12. Therefore, increased activation of AKT via LSC interaction with cryptic collagen IV epitopes in leukemic BM may promote leukemogenesis in addition to regulating migration.

**Figure 2:**
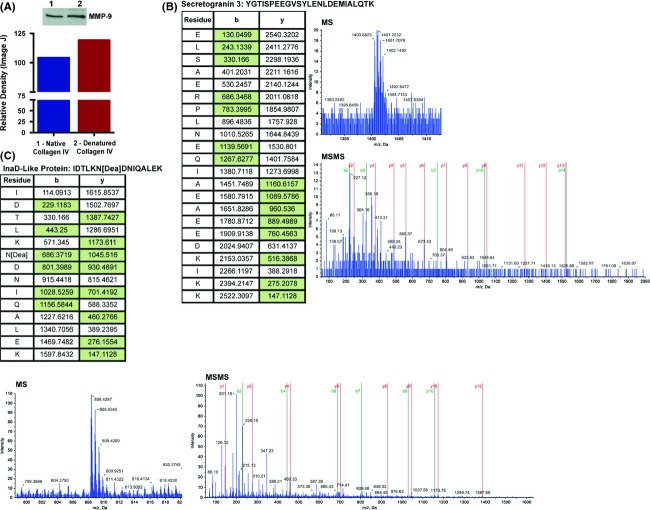
Cryptic collagen IV exposure increases MMP-9 and other secreted factors in K562 cells. (A) MMP-9 expression in the CM was determined using Western blot. Respective pixel densities were graphed. (B and C) Representative time-of-flight mass spectra (MS, top right panel in B and bottom left panel in C) and collision-induced sequence data for selected high confidence (>98%) MS peptides (MSMS lower right panels) for Secretogranin (B) and InaD-like protein (C) in denatured collagen IV conditioned media from LC-MS-MS QSTAR proteomic analysis. For clarity of reference to MSMS data, only selected critical y + 1 ion and b + 1 ion series are shown in green shade (Tables, left panels).

Analysis of secreted matrix metalloproteinases 9 (MMP-9) in the CM via western blot from K562 cells exposed to either native or denatured collagen IV showed increased levels of MMP-9 with denatured collagen IV (Fig. 2A). Interestingly, MMP-9 dependent exposure of the HUIV26 cryptic site on collagen IV has been showed to promote endothelial cell migration and neovascularization 13. Currently, these outcomes suggest a possible feed forward loop for collagen IV-DDR1-MMP9-cryptic collagen IV in promoting migration and adhesion of leukemic cells. Further, liquid chromatography/tandem mass spectrometry (LC/MS/MS) QSTAR proteomic analysis of CM revealed exclusive presence of Secretogranin 3 (SCG3/SGIII) and InaD-like protein (INADL/PATJ) in the denatured collagen IV CM (Fig. 2B–C) in addition to several other interesting factors (top ones shown in Table S1). INADL/PATJ is a multiple PDZ domain containing protein known to localize to tight junctions or apical membranes and is predicted to regulate cell polarity 14. SCG3/SGIII belongs to the granin family and is known to regulate secretory pathways of peptides, hormones, neurotransmitters, and growth factors in the transgolgi-network 15. These novel cryptic collagen IV-induced factors in the CM of leukemic cells may play supportive roles in promoting migration and adhesion in BM niche.

**Figure 3:**
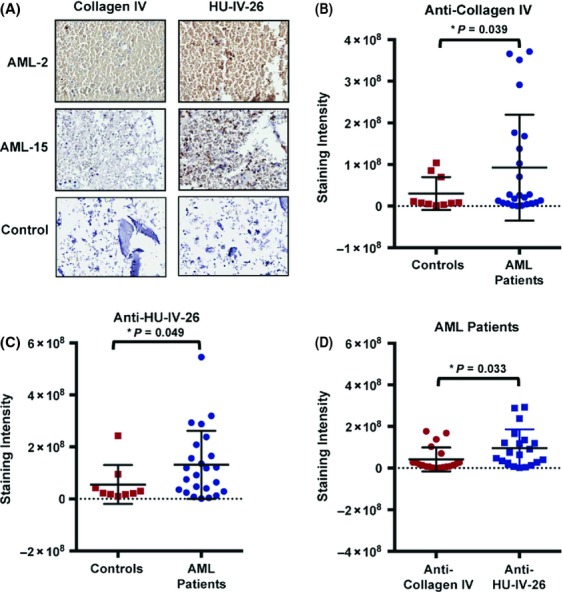
Expression of cryptic collagen IV is increased in BM samples of AML samples. (A) Representative immunohistochemistry images of AML and normal control bone marrow (BM) aspirates. (B) Anti-Collagen IV staining intensity in normal control BM aspirates (*n* = 10) and AML patients (*n* = 24) (C) Anti-HU-IV-26 staining intensity in same samples as B. (D) Anti-Collagen IV and Anti-HU-IV-26 staining intensity of *n* = 20 of the 24 AML patient samples.

Lastly, we examined levels of native collagen IV and remodeled collagen IV in BM-derived leukemic blasts of AML patients (*n* = 24, patient information provided in Table S2) using HUIV26 and a native collagen IV antibody via TMA with IHC. In comparison to control samples (*n* = 10), native and HUIV26 cryptic collagen IV epitope were found in significantly high levels in patient samples (Fig. 3A–C). Most importantly, the levels of the HUIV26 epitope were significantly higher than native collagen IV in AML patients (Fig. 3D). Notably, the DDR1 levels (as previously reported) were concurrently elevated in the same AML patients with high HUIV26 epitope as examined via TMA and IHC 2. Association of leukemic blasts and LSCs with ECM components and stromal cells via enhanced adhesion is known to generate proliferative and survival signals required for their sustenance in endosteal region of BM niche 16. Importantly, adhesion inhibitors like plerixafor and MDX-1338 (anti-CXCR4 antibody) in combination with standard induction chemotherapy are being evaluated (ongoing multicenter phase I trial) for their ability to mobilize LSCs from BM niche 16. Given our findings of increased adhesion of leukemic cells to remodeled collagen IV, further characterization of the remodeled collagen-binding partners on leukemic cells may facilitate the identification and development of novel mobilizing drugs, which may eventually help in improving outcomes and efficiency of AML therapy.

**Figure 4:**
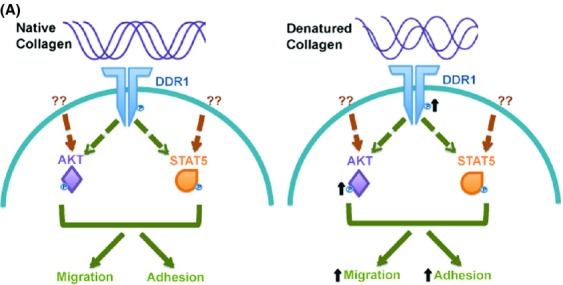
Hypothesized model where cryptic collagen IV promotes cell migration and adhesion in myeloid leukemia. (A) Hypothesized model for cryptic collagen IV activation of DDR1 and AKT-promoting cell migration and adhesion in myeloid leukemia illustrating proposed signaling pathway and mechanistic roles of myeloid leukemia cells upon interaction with either native or denatured collagen.

In summary, for the first time, we demonstrate that remodeled collagen IV is a potent activator of DDR1 and AKT that also modulates both migration and adhesion of myeloid leukemia cells (Fig. 4). Additionally, high levels of the HUIV26 cryptic collagen IV epitope are expressed in BM of AML patients. Taken together, further understanding of this phenomenon may lead to the development of therapeutic agents that directly modulate the BM microenvironment and attenuate leukemogenesis.

## Supporting Information

Figure S1 DDR1 expression was examined using flow cytometry. Histogram overlay shows the relative DDR1 expression (red – K562 cells, purple – NB4 cells and green – HL-60).

Table S1 MS/MS data for selected high confidence (>95%) peptides.

Table S2 Clinicopathological information of the AML tissue microarray specimens analyzed by IHC analysis.
