# Supplementary figures and images for: Cryptic collagen IV promotes cell migration and adhesion in myeloid leukemia
Source: Cancer Med. 2014 Feb 12;3(2):265–72. doi: 10.1002/cam4.203 (PMC3987076; doi:10.1002/cam4.203)

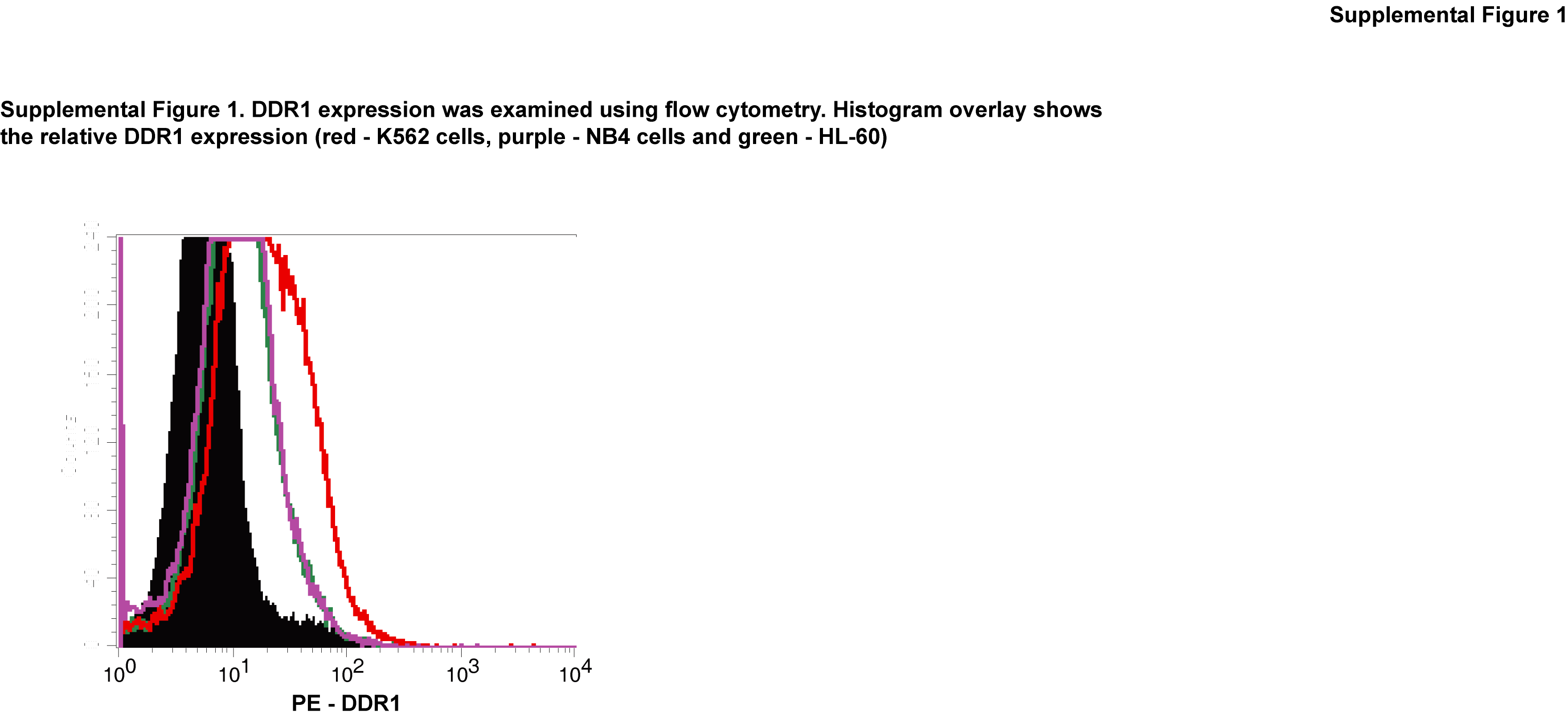

Supplement: Figure S1 — DDR1 expression was examined using flow cytometry. Histogram overlay shows the relative DDR1 expression (red – K562 cells, purple – NB4 cells and green – HL-60). [file cam40003-0265-sd1.tif]
