# Supplementary material for: Cryptic collagen IV promotes cell migration and adhesion in myeloid leukemia
Source: Cancer Med. 2014 Feb 12;3(2):265–72. doi: 10.1002/cam4.203 (PMC3987076; doi:10.1002/cam4.203)
Supplement: Table S1 — MS/MS data for selected high confidence (>95%) peptides. [file cam40003-0265-sd2.pdf]

Native Collagen IV Conditioned Media with K562 Cells			
**Accession #**	**Name**	**Species**	**Peptides(95%)**
sp|P00441|SODC_HUMAN	Superoxide dismutase [Cu-Zn] OS=Homo sapiens GN=SOD1 PE=1 SV=2	HUMAN	5
sp|P62328|TYB4_HUMAN	Thymosin beta-4 OS=Homo sapiens GN=TMSB4X PE=1 SV=2	HUMAN	3
sp|P02768|ALBU_HUMAN	Serum albumin OS=Homo sapiens GN=ALB PE=1 SV=2	HUMAN	6
sp|P02649|APOE_HUMAN	Apolipoprotein E OS=Homo sapiens GN=APOE PE=1 SV=1	HUMAN	2
sp|P60174|TPIS_HUMAN	Triosephosphate isomerase OS=Homo sapiens GN=TPI1 PE=1 SV=3	HUMAN	1
sp|P62937|PPIA_HUMAN	Peptidyl-prolyl cis-trans isomerase A OS=Homo sapiens GN=PPIA PE=1 SV=2	HUMAN	2
sp|P17096|HMGA1_HUMAN	High mobility group protein HMG-I/HMG-Y OS=Homo sapiens GN=HMGA1 PE=1 SV=3	HUMAN	0
sp|Q96QG7|MTMR9_HUMAN	Myotubularin-related protein 9 OS=Homo sapiens GN=MTMR9 PE=1 SV=1	HUMAN	0
sp|Q96I13|ABHD8_HUMAN	Abhydrolase domain-containing protein 8 OS=Homo sapiens GN=ABHD8 PE=2 SV=1	HUMAN	0
sp|O14646|CHD1_HUMAN	Chromodomain-helicase-DNA-binding protein 1 OS=Homo sapiens GN=CHD1 PE=1 SV=2	HUMAN	0
sp|Q8NDV7-6|TNR6A_HUMAN	Isoform 6 of Trinucleotide repeat-containing gene 6A protein OS=Homo sapiens GN=TNRC6A	HUMAN	0

Denatured Collagen IV Conditioned Media with K562 Cells			
**Accession #**	**Name**	**Species**	**Peptides(95%)**
sp|P06733|ENOA_HUMAN	Alpha-enolase OS=Homo sapiens GN=ENO1 PE=1 SV=2	HUMAN	5
sp|Q8WXD2|SCG3_HUMAN	Secretogranin-3 OS=Homo sapiens GN=SCG3 PE=1 SV=3	HUMAN	4
sp|P02768|ALBU_HUMAN	Serum albumin OS=Homo sapiens GN=ALB PE=1 SV=2	HUMAN	13
sp|P00441|SODC_HUMAN	Superoxide dismutase [Cu-Zn] OS=Homo sapiens GN=SOD1 PE=1 SV=2	HUMAN	4
sp|P62937|PPIA_HUMAN	Peptidyl-prolyl cis-trans isomerase A OS=Homo sapiens GN=PPIA PE=1 SV=2	HUMAN	1
sp|P02771|FETA_HUMAN	Alpha-fetoprotein OS=Homo sapiens GN=AFP PE=1 SV=1	HUMAN	1
sp|P60174|TPIS_HUMAN	Triosephosphate isomerase OS=Homo sapiens GN=TPI1 PE=1 SV=3	HUMAN	1
sp|Q8NI35|INADL_HUMAN	InaD-like protein OS=Homo sapiens GN=INADL PE=1 SV=3	HUMAN	1
sp|Q9H2D6|TARA_HUMAN	TRIO and F-actin-binding protein OS=Homo sapiens GN=TRIOBP PE=1 SV=3	HUMAN	0
sp|P08572|CO4A2_HUMAN	Collagen alpha-2(IV) chain OS=Homo sapiens GN=COL4A2 PE=1 SV=4	HUMAN	2
sp|O43603|GALR2_HUMAN	Galanin receptor type 2 OS=Homo sapiens GN=GALR2 PE=1 SV=1	HUMAN	0
