# Supplementary material for: Cryptic collagen IV promotes cell migration and adhesion in myeloid leukemia
Source: Cancer Med. 2014 Feb 12;3(2):265–72. doi: 10.1002/cam4.203 (PMC3987076; doi:10.1002/cam4.203)
Supplement: Table S2 — Clinicopathological information of the AML tissue microarray specimens analyzed by IHC analysis. [file cam40003-0265-sd3.pdf]

Manuscript ID	Sample ID	Sex	Specimen Type	FAB	Final Diagnosis	Clinical History	Comments
AML-01	RA96-0006	Female	Acute Myelogenous Leukemia	M1	Acute Myeloblastic Leukemia (M1)	Note: Bone marrow aspirate original tube broke. Broken tube with clot sent as well as fluid aspirate.	N/A
AML-02	RA96-0007	Female	Acute Myelogenous Leukemia	M1	Acute Myeloblastic Leukemia (M1)	N/A	N/A
AML-03	RA96-0008	Female	Acute Myelogenous Leukemia	M2	Acute Myeloblastic Leukemia (M2)	Lymphoma - AML	A flow cytometry performed on the marrow aspirate material shows a population of immature myeloid cells consistent with acute myeloblastic leukemia. No lymphoma is seen.
AML-04	RA96-0009	Female	Acute Myelogenous Leukemia	M2	Acute Myelogenous Leukemia (M2), See Comment	Follow-up lymphoma = AML	Flow cytometric studies of peripheral blood and bone marrow demonstrate a population of cells consistent with myeloblasts. There is no evidence of T lymphoblastic lymphoma by cytometric studies.
AML-05	RA96-0010	Male	Acute Myelogenous Leukemia	M4	Acute Myelomonocytic Leukemia (M4)	R/O AML	N/A
AML-06	RA96-0011	Male	Acute Myelogenous Leukemia	M4	Acute Myelomonocytic Leukemia (M4)	F/U myelodysplasia	The combination of both morphology and cytochemical stains support diagnosis of acute myelomonocytic leukemia. If the percentage of monocytic cells were slightly higher (80%) this leukemia would be better classified as acute monocytic leukemia.
AML-07	RA96-0013	Female	Acute Myelogenous Leukemia	N/A	Persistent Acute Myelogenous Leukemia	Acute Leukemia	N/A
AML-08	RA96-0014	Female	Acute Myelogenous Leukemia	N/A	Persistent Acute Promyelocytic Leukemia	N/A	N/A
AML-09	RA96-0015	Male	Acute Myelogenous Leukemia	N/A	Persistent Acute Myelomonocytic Leukemia	AML Day No. 25. R/O regeneration; R/O remission	Most of the cells are early blast forms consistent with persistence or relapse of this patient's acute myelomonocytic leukemia.
AML-10	RA96-0016	Female	Acute Myelogenous Leukemia	N/A	Persistent Acute Myeloblastic Leukemia	N/A	The overwhelming majority of the cells present are blasts as seen in the patient's previous bone marrows.
AML-11	RA96-0017	Female	Acute Myelogenous Leukemia	N/A	Persistent Acute Myelomonocytic Leukemic Infiltrate	N/A	There is a large population of blasts present, consistent with persistent leukemic infiltrate. Some of these have a monocytoid appearance.
AML-12	RA96-0018	Female	Acute Myelogenous Leukemia	N/A	Hypocellular bone marrow showing effects of chemotherapy with Persistent Acute Myeloblastic Leukemic Infiltrate	AML following Chemo	N/A

AML-13	RA96-0019	Female	Acute Myelogenous Leukemia	N/A	Persistent Acute Myeloblastic Leukemic Infiltrate	Acute myeloid leukemia. Day #11	Most of the cells present are immature myeloid cells consistent with persistent acute leukemic infiltrate.
AML-14	RA99-0035	Female	AML FAB M2	M2	Acute Myelogenous Leukemia, most consistent with FAB-M2 morphology.	AML	These combined features are indicative of acute myelogenous leukemia and are most consistent with FAB-M2 morphology.
AML-15	RA99-0063	Female	AML FAB M1	M1	Acute Myelogenous Leukemia, most consistent with FAB-M1 morphology.	History of lymphoma; now with pancytopenia.	This immunophenotype in conjunction with the above morphology is indicative of acute myelogenous leukemia, most consistent with FAB-M1 morphology.
AML-16	RA99-0067	Male	AML	N/A	Slight atypical plasmacytosis.	6 months after dx - multiple myeloma. On M2.	If this patient has previously established diagnosis for myeloma, the plasma cells clustered together in this biopsy most likely represent residual disease.
AML-17	RA99-0138	Female	AML	N/A	Relapsed Acute Myelogenous Leukemia	Follow-up AML	Composite features are indicative of relapsed acute leukemia.
AML-18	RA99-0166	Female	AML FAB M1	M1	Acute Myelogenous Leukemia, most consistent with FAB-M1 morphology.	Leukopenia, R/O Leukemia	The composite features are indicative of acute myelogenous leukemia, most consistent with FAB-M1 morphology.
AML-19	RA99-0192	Female	AML	N/A	Relapsed Acute Myeloblastic Leukemia	AML relapse	N/A
AML-20	RA99-0197	Female	AML FAB M4	M4	Acute Myeloblastic Leukemia (M4)	Acute myeloid leukemia.	The findings are consistent with a diagnosis of acute myeloblastic leukemia (M4).
AML-21	RA98-1581	Female	AML	N/A	Persistent Acute Myelogenous Leukemia	AML, Day #13, R/O Aplasia	Chromosomal studies performed at _____ demonstrate a normal female karyotype (46.XX)
AML-22	RA98-1582	Male	AML	N/A	Acute Myeloblastic Leukemia arising in a setting of Myelodysplasia.	MDS with leukocytosis	Compared to the previous bone marrow obtained in _____, there are now more recognizable blasts. The other dysplastic features persist.
AML-23	RA98-1589	Female	AML	N/A	Mononuclear cell infiltrate consistent with relapsing Acute Myeloblastic Leukemia	MDS	N/A
AML-24	RA99-0062	Female	AML	N/A	Interstital, immature mononuclear cell infiltrate, consistent with Acute Myelogenous Leukemia. --- Atypical lymphoid infiltrates, suspicious for small cell lymphoproliferative disorder.	287.5 AML (Bone marrow slides to Dr. ________)	Features are consistent with Acute Myelogenous Leukemia, M2 morphology. The atypical lymphoid aggregates are suspicious for a small cell lymphoproliferative disorder. Correlation with clinical history, flow cytometric immunophenotyping, and bone marrow cytogenetics is suggested.
Control-1	RA96-0074	Male	Normal Sample	Normal Sample	Normocellular bone marrow with maturing hematopoiesis; no neoplasm seen.	F/U Lymphoma	N/A
Control-2	RA96-0075	Male	Normal Sample	Normal Sample	Normocellular bone marrow with maturing, trilineage hematopoiesis; no neoplasm seen.	R/O Lymphoma	N/A

Control-3	RA96-0077	Male	Normal Sample	Normal Sample	Normocellular bone marrow showing trilineage hematopoiesis and increased storage iron; no neoplasm seen.	Lymphoma	N/A
Control-4	RA96-0078	Female	Normal Sample	Normal Sample	Adequately cellular bone marrow showing trilineage hematopoiesis and decreased storage iron; no neoplasm seen.	None given.	N/A
Control-5	RA96-0079	Female	Normal Sample	Normal Sample	Normocellular bone marrow with increased storage iron; no neoplasm seen.	Lymphoma	N/A
Control-6	RA96-0080	Female	Normal Sample	Normal Sample	Adequately cellular bone marrow with trilineage hematopoiesis and increased storage iron.	ET	N/A
Control-7	RA96-0082	Male	Normal Sample	Normal Sample	Normocellular bone marrow, no neoplasm seen.	Lymphoma	N/A
Control-8	RA96-0083	Female	Normal Sample	Normal Sample	Persistent involvement by small cell lymphoproliferative disorder.	CLL	N/A
Control-9	RA96-0076	Female	Normal Sample	Normal Sample	Normocellular bone marrow with maturing, trilineage hematopoiesis; no neoplasm seen.	R/O Myeloma	N/A
